# Wireless Channel Propagation Characteristics and Modeling Research in Rice Field Sensor Networks

**DOI:** 10.3390/s18093116

**Published:** 2018-09-15

**Authors:** Zhenran Gao, Weijing Li, Yan Zhu, Yongchao Tian, Fangrong Pang, Weixing Cao, Jun Ni

**Affiliations:** National Engineering and Technology Center for Information Agriculture, Jiangsu Key Laboratory for Information Agriculture, Key Laboratory for Crop System Analysis and Decision Making, Ministry of Agriculture, Jiangsu Key Laboratory for Information Agriculture, Jiangsu Collaborative Innovation Center for the Technology and Application of Internet of Things, Nanjing Agricultural University, Nanjing 210095, China; gzrsunrise@163.com (Z.G.); 2016101036@njau.edu.cn (W.L.); yanzhu@njau.edu.cn (Y.Z.); yctian@njau.edu.cn (Y.T.); pangfangrong@njau.edu.cn (F.P.); caow@njau.edu.cn (W.C.)

**Keywords:** rice fields, propagation characteristics, path loss, two-slope logarithmic model

## Abstract

Wireless channel propagation characteristics and models are important to ensure the communication quality of wireless sensor networks in agriculture. Wireless channel attenuation experiments were carried out at different node antenna heights (0.8 m, 1.2 m, 1.6 m, and 2.0 m) in the tillering, jointing, and grain filling stages of rice fields. We studied the path loss variation trends at different transmission distances and analyzed the differences between estimated values and measured values of path loss in a free space model and a two-ray model. Regression analysis of measured path loss values was used to establish a one-slope log-distance model and propose a modified two-slope log-distance model. The attenuation speed in wireless channel propagation in rice fields intensified with rice developmental stage and the transmission range had monotone increases with changes in antenna height. The relative error (RE) of estimation in the free space model and the two-ray model under four heights ranged from 6.48–15.49% and 2.09–13.51%, respectively, and these two models were inadequate for estimating wireless channel path loss in rice fields. The ranges of estimated RE for the one-slope and modified two-slope log-distance models during the three rice developmental stages were 2.40–2.25% and 1.89–1.31%, respectively. The one-slope and modified two-slope log-distance model had better applicability for modeling of wireless channels in rice fields. The estimated RE values for the modified two-slope log-distance model were all less than 2%, which improved the performance of the one-slope log-distance model. This validates that the modified two-slope log-distance model had better applicability in a rice field environment than the other models. These data provide a basis for modeling of sensor network channels and construction of wireless sensor networks in rice fields. Our results will aid in the design of effective rice field WSNs and increase the transmission quality in rice field sensor networks.

## 1. Introduction

Wireless sensor networks (WSNs) are composed of many sensor nodes in self-organization and multi-hop modes. In agriculture, these networks can provide real-time and efficient acquisition of environmental and crop data. They can effectively decrease labor requirements and reduce the costs of agricultural production [1,2]. However, WSNs have limited node resources. As signal transmission paths between transmitter ends and receiver ends in WSN, wireless channels are easily affected by terrain, crop height, crop density, field obstacles, and node layout of the farm environment [3]. Darr et al. [4] used empirical testing methods to study path loss in poultry farms. Poultry cages and concrete floor separations resulted in channel path loss and attenuation. Rizman et al. [5] analyzed the variation patterns on signal intensity and power loss rate in ZigBee sensor nodes in vineyards and oil palm plantations. The leaves and fruits caused scattering and absorption and accelerated path loss and attenuation in wireless channel networks. Hebel et al. [6] studied signal attenuation and intensity under direct line of sight paths on flat grass patches. Distance and height were determining factors affecting path loss. The widespread application of WSNs in farmland environments will depend on clarifying the factors that affect the model precision of path loss in wireless channels in farmland environments, predicting the configuration height and coverage range of WSN nodes, and ensuring transmission quality of WSNs [7].

Many studies have evaluated the channel propagation characteristics and modeling of WSNs [8,9,10]. Shen et al. [11] set up three antenna heights in a field (25 cm, 45 cm, and 1 m) to test the propagation characteristics of 433 MHz wireless signals. A one-slope loss model was judged to be superior as a low-altitude propagation model for wireless channels. Liu et al. [12,13] carried out 2.4 GHz wireless signal propagation performance experiments in a wheat field at different growth stages. The wireless channel path loss at different wheat heights conformed to a log-distance path loss model with plant height and canopy coverage being the main factors involved. Zhang et al. [14] compared four wireless channel path loss models in a wheat field and proposed a modified two-slope log-distance model. These models can be used for simulation of signal attenuation of WSNs in different transmission environments. However, there are significant differences between channel loss models for WSNs in different environments [15,16]. In rice fields, different growth stages can have different water requirements. This causes the wireless transmission environment to be more complex than in wheat fields. However, there are no reports on path loss regularity of wireless channels in rice fields. Therefore, there is a need to evaluate channel loss models in different rice field environments, analyze the applicability of different propagation loss models, and construct wireless channel path loss models suitable for rice. This information will aid in the design of effective rice field WSNs and increase the transmission quality in rice field sensor networks.

We conducted field tests to determine the received power of wireless signals at different rice developmental stages, different transmission distances, and different antenna heights. We analyzed wireless channel propagation characteristics in rice fields and clarified the propagation loss laws and coverage area of nodes in wireless channels. We analyzed the applicability of four wireless channel models, studied the main factors affecting wireless channel propagation characteristics, and constructed 2.4 GHz wireless channel propagation loss models for rice fields. The data supports the engineering, application and networking of WSNs in rice fields.

## 2. Materials and Methods

### 2.1. Experimental Equipment

The transmitter end and receiver end were the sensor and sink nodes of rice field WSNs. The sensor nodes of rice field WSNs can measure soil profile moisture, crop canopy moisture, and field water level information. The sink node is used to process rice field moisture data and for network transmission. The embedded XBee-PRO S2B module from Digi International Inc. (Minnetonka, MN, USA) was used as wireless transmission Zigbee modules for the sensor and sink nodes. The primary characteristic indicators for the Zigbee module were: frequency: 2.4 GHz, modulation technique: DSSS, baud rate: 250 KB/S, data throughput: 3500 bps, transmission power: 18 dBm, and receiver sensitivity: −102 dBm. To increase the transmission ability of wireless signals, external vertically polarized omnidirectional antennas were used for the transmitter and receiver ends [4] and the antenna gain was 2.1 dBi and 18 cm long. A serial port connected computer was used for the receiver end of the WSN and the XCTU software (Digi International Inc.) was used to achieve transmitter end parameter control and acquisition and the recording of received signal strength indication (RSSI) at the receiver end.

### 2.2. Experiment Design

**Figure 1:**
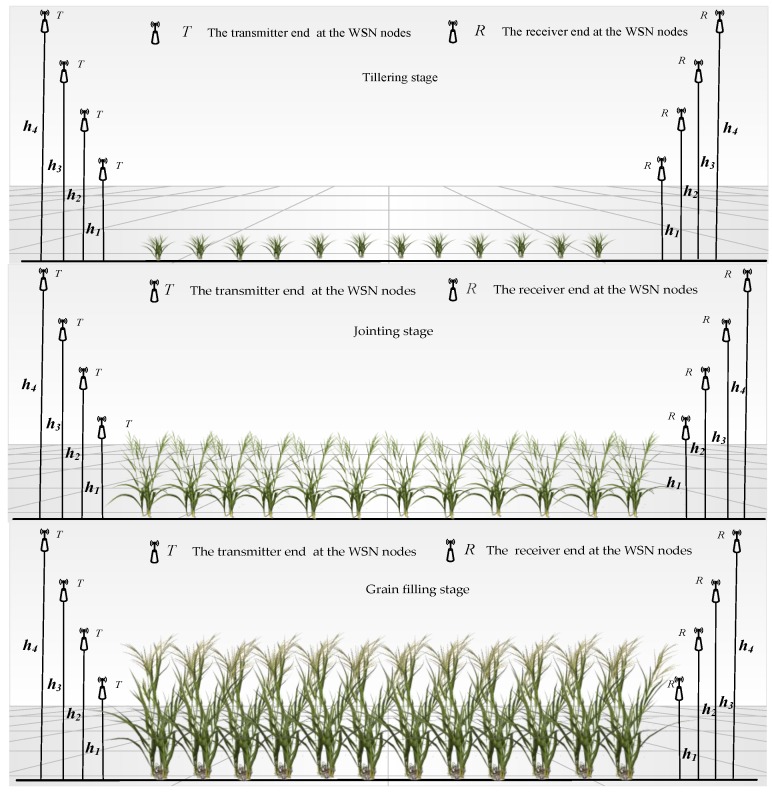
Experiment design of the three scenarios.

To analyze the path loss characteristics of wireless channels in rice fields and minimize terrain and weather effects on the results, the experiment was conducted in a flat rice field on sunny days. Testing took place from July to September 2016 at the Rugao experiment demonstration base in the National Engineering and Technology Center for Information Agriculture (Baipu Town, Rugao City, Jiangsu Province, China, 120°19′ E, 32°14′ N). The experimental rice field had an area of 600 × 100 m^2^ on flat ground. Rice was planted using conventional row and plant spacing, with an inter-row spacing of 0.3 m and an inter-plant spacing of 0.4 m. During transplantation to maturity, plant height changed from 0.2 to 1.2 m and canopy coverage changed from 10 to 90%. The three growth stages (tillering stage, jointing stage, and grain filling stage) with large changes in rice plant height and canopy coverage and higher requirements for moisture perception and intelligent irrigation were the three experimental treatments. At the end of the tillering stage, the mean plant height was 0.2 m, canopy coverage was <45%, and coverage was low. During the jointing stage, the mean plant height was 0.6 m, canopy coverage ranged from 45%–75%, and coverage was moderate. During the grain filling stage, the mean plant height was 1.1 m, canopy coverage was >75%, and cover type was high. The height of the transmitter and receiving antennas were set as h_1_ (0.8 m), h_2_ (1.2 m), h_3_ (1.6 m), and h_4_ (2.0 m) according to the variation characteristics of plant height and canopy coverage of rice in the developmental stages. Figure 1 shows the experiment design of the three treatments. In Figure 1, there were no tall buildings or other obstacles between the transmitter end and receiver end at the WSN nodes and antennas for all nodes were placed vertically to eliminate the effects of antenna polarity on results. The receiver end R was fixed at the signal source and used as a starting point. RSSI test points were placed at 7 m intervals along a straight line from the receiver end, with a total of 70 dynamic test points. The heights of the antennas at the receiver end R and the transmitter end T of every test point were the same and the greatest distance between the transmitter and receiver was 490 m.

### 2.3. Test Items

**Figure 2:**
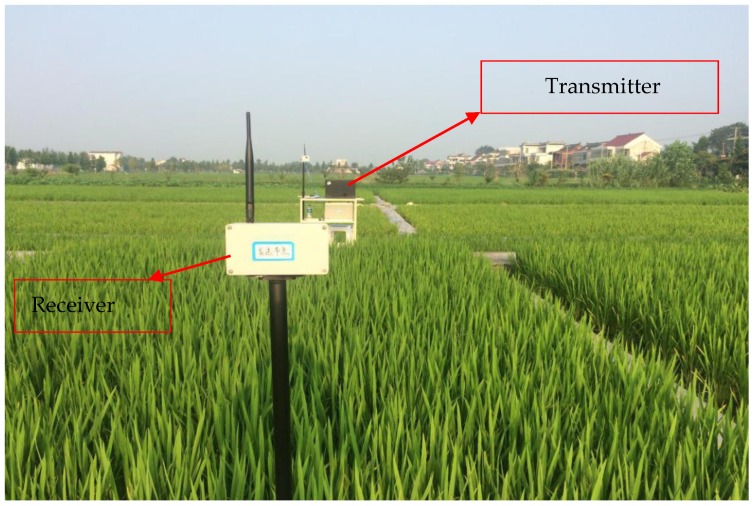
Field experiment site map.

The test indicators for wireless channel propagation characteristic experiments in rice fields were receiver end signal power at different developmental stages (tillering stage, jointing stage, and grain filling stage). These were the RSSI values of received signals. During the experiment, pole supports with variable heights were used to elevate the transmitter and receiver ends at a 0.8 m height above the ground. The receiver end was fixed at the start point. Following that, ten 34-bit long data packets were sent at dynamic test points at 7 m intervals according to the experimental design. The receiver end and computer XCTU software (ver. 5.2.8.6, Digi International Inc, Minnetonka, MN, USA) was used to acquire RSSI values and the mean RSSI values were stored. Figure 2 shows the field experiment map. After h_1_ experiments were completed, the pole height was modified by changing the node elevations to 1.2 m, 1.6 m, and 2.0 m. RSSI values were obtained at these heights while other conditions remained unchanged. Triplicate experiments were carried out at for these three scenarios.

## 3. Data Analysis and Methods

### 3.1. Calculation of Path Loss

The channel path loss $P_{L\left(d\right)}$ was calculated and generated using RSSI values [17]. From this, the connection quality was calculated using the following formula:

(1) $$RSSI=10\mathrm{lg}P_{r}{}_{\left(d\right)},$$

where Pr(d) (dBm) is the receiver power at distance d and the calculation formula for channel path loss $P_{L\left(d\right)}$ is:

(2) $$P_{L}{}_{\left(d\right)}=p_{t}+G_{t}+G_{r}-P_{r}{}_{\left(d\right)},$$

where Pt (dBm) is transmitter power, Gt (dBi) is transmitting antenna gain, and Gr (dBi) is the receiving antenna gain. According to the equipment parameters, Pt was 18 dBm, and transmitting antenna gain Gt and receiving antenna gain Gr were both 2.1 dBi.

### 3.2. Path Loss Models

#### 3.2.1. Free Space Model

The path loss in the free space model [18] is under ideal propagation conditions and the model was constructed under ideal conditions in which path loss is only related to wireless signal frequency and propagation distance. This model only considers the effects of frequency and propagation distance on wireless channels in the rice field sensor network and does not consider the presence of rice between the wireless signal transmitter end and receiver end. It does not consider the effects of reflection, refraction, diffraction, and absorption of signals by rice. In this paper, both the transmitting end and the receiving end use U.FL antenna to Sub-Miniature-A vertically polarized omnidirectional antenna with power gain *G_r_* = *G_t_* = 2.1 dBi. Therefore, the formula for calculating the received signal power from the transmitting end *d* in free space [18] is *P_r_(d)* as follows:

(3) $$P_{r}{}_{\left(d\right)}=p_{t}G_{t}G_{r}{\left(\frac{\lambda }{4\pi d}\right)}^{2}.$$

The path loss $P_{L}\left(d\right)$ calculation formula is:

(4) $$P_{L}{}_{\left(d\right)}=10\mathrm{lg}\left(\frac{P_{t}}{P_{r}{}_{\left(d\right)}}\right).$$

The formula for calculating the free space path loss model obtained by substituting Equation (3) into Equation (4) is:

(5) $$P_{L}{}_{\left(d\right)}=20\mathrm{lg}\left(4\pi \right)+20\mathrm{lg}d-20\mathrm{lg}\lambda -10\mathrm{lg}G_{t}-10\mathrm{lg}G_{r}.$$

In this equation, λ represents the wavelength of the wireless signal and d is the distance between the receiver end and transmitter end.

#### 3.2.2. Two-Ray Model

The two-ray model [19] considers the effects of direct waves and ground reflected waves on signal propagation. There are path differences between these two waves and the reflection distance under different antenna heights are different. This produces additional phase shifts and results in attenuation of the combined wave. This model only considers the effects of direct waves between the transmitter end and receiver end and reflected waves at the canopy layer of rice fields on wireless channels in the rice field sensor network and does not consider the effects of other factors. The formula for the path loss in the two-ray model is as follows:

(6) $$P_{L}{}_{\left(d\right)}=40\mathrm{lg}d-20\mathrm{lg}h_{t}-20\mathrm{lg}h_{r}-10\mathrm{lg}G_{t}-10\mathrm{lg}G_{r},$$

where $h_{t}$ is the height of the transmitting antenna and $h_{r}$ is the height of the receiving antenna. *G_r_* = *G_t_* = 2.1 dBi.

#### 3.2.3. One-Slope Log-Distance Model

The one-slope log-distance model [20] expresses path loss as a linear function of the logarithmic distance and the path loss index is used to express the speed of channel path loss. Due to the presence of direct transmission, reflection and refraction in the channels, the received signals contain large numbers of plane waves with random amplitudes, phases and angles of arrival, resulting in distortion or attenuation of the received signals. This model considers the changes due to changes in plant height and canopy coverage in rice plants in wireless channels in the rice field sensor network. These changes result in direct transmission, reflection, and refraction of channels simultaneously occurring and affecting transmission of channel signals. The formula for the path loss based on the one-slope log-distance model is as follows:

(7) $$P_{L}{}_{\left(d\right)}=40\mathrm{lg}d-20\mathrm{lg}h_{t}-20\mathrm{lg}h_{r}-10\mathrm{lg}G_{t}-10\mathrm{lg}G_{r},$$

where $d_{0}$ is the reference propagation distance, $P_{L}{}_{\left(d_{0}\right)}$ is the path loss in the reference propagation distance $d_{0}$, *n* is the path loss factor, and $X_{\sigma }$ is the Gaussian random variable that is the standard deviation σ caused by shadow fading. $P_{L}{}_{\left(d_{0}\right)}$ is related to the specific application environment, with a general reference distance $d_{0}$ of 1 m. $P_{L0}$ is used to express the initial loss at the reference distance of 1 m. Equation (7) can be simplified as

(8) $$P_{L}{}_{\left(d\right)}=K+10n\mathrm{lg}d,$$

where *K* is the sum of $P_{L}{}_{\left(d_{0}\right)}$ and $X_{\sigma }$. The path loss factor, *n*, of the rice field sensor network is related to the height of rice plants, canopy coverage, and surrounding environment. The values of *n* and *K* are obtained by regression analysis of measured values using the least squares method [21,22].

#### 3.2.4. Modified Two-Slope Log-Distance Model

Breakpoints exist in surface wireless channel propagation and these channels are divided into two different propagation spaces that directly affect the quality of channel modeling [23]. Before the breakpoint, signals undergo slow attenuation with distance, but, after the breakpoint, signal attenuation is rapid as distances increase further [24]. The one-slope log-distance model does not consider the effects of breakpoints on model precision. Therefore, in order to improve the modeling performance of this method, we employed a two-slope log-distance model with two intermittent lines for modeling of wireless channel path loss. The formula for the path loss for the modified two-slope log-distance model is as follows:

(9) $$P_{L}{}_{\left(d\right)}=\{\begin{array}{cc} K_{2}+10n_{2}\mathrm{lg}d & \left(d>d_{b}\right)\\ K_{1}+10n_{1}\mathrm{lg}d & \left(d\leq d_{b}\right) \end{array},$$

where *K*_1_ and *K*_2_ represent the parameters of the two-slope log-distance model. *K*_1_ is the summation of the initial path loss before the breakpoint and the Gaussian random variable σ. *K*_2_ is the summation of the initial path loss before the breakpoint and the Gaussian random variable σ. *n*_1_ and *n*_2_ represent the path loss factors before and after the breakpoint, respectively. db represents the distance between the transmitter end and the receiver end at the breakpoint. The fitting rules of the least squares method were used for linear fitting of the measured loss so that the residual sum of squares and Q(*b*, *n*_1_, *n*_2_) were the smallest; then,

(10) $$\begin{array}{l} Q\left(b,n_{1},n_{2}\right)={\sum_{i=1}^{b}\left[P_{L}{}_{\left(d_{i}\right)}-P_{L}{}_{\left(d_{b}\right)}-10n_{1}\mathrm{lg}\left(d_{i}/d_{b}\right)\right]}^{2}+\\ \begin{array}{ccc} & & {\sum_{i=b+1}^{m}\left[P_{L}{}_{\left(d_{i}\right)}-P_{L}{}_{\left(d_{b+1}\right)}-10n_{2}\mathrm{lg}\left(d_{i}/d_{b}\right)\right]}^{2} \end{array} \end{array}$$

Here, $Q\left(b,n_{1},n_{2}\right)$ is the function of b, *n*_1_, and *n*_2_. Adjustment of these three parameters was used to minimize $Q\left(b,n_{1},n_{2}\right)$. As *b* is the location of the unknown breakpoint, the 2nd to 70th propagation distance points were transversed during regression to identify the residual sum of squares and smallest distance points in the two slopes [24]. When $Q\left(b,n_{1},n_{2}\right)$ was the smallest, *b* was taken as the location of the breakpoint. This was used to confirm the model parameters and construct the two-slope log-distance model.

### 3.3. Model Evaluation Standards

The root-mean-square error (RMSE) and relative error (RE) were used as precision indicators for predicted path loss in the models. As these values approach 0, the prediction precision increases. The calculation formula is as follows:

(11) $$RMSE=\sqrt{\frac{1}{n}\times \sum_{i=1}^{n}{\left(P_{L\left(di\right)}-{\hat{P}}_{L\left(di\right)}\right)}^{2}},$$

(12) $$RE=\sqrt{\frac{1}{n}\times \sum_{i=1}^{n}{\left(\frac{P_{L\left(di\right)}-{\hat{P}}_{L\left(di\right)}}{{\hat{P}}_{L\left(di\right)}}\right)}^{2}}.$$

Here, *n* represents the number of test samples in the experiment.

## 4. Results and Analysis

### 4.1. Analysis of Variation Trends of Wireless Channel Propagation in Rice Fields

**Figure 3:**
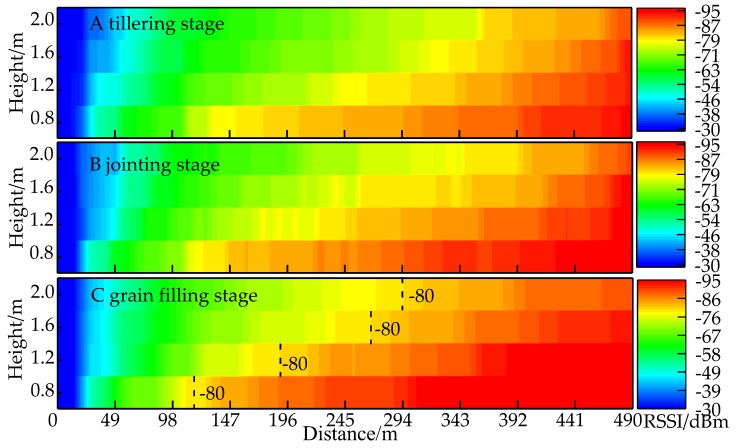
Corresponding channel path loss at different antenna heights in the (**A**) tillering stage; (**B**) jointing stage; and (**C**) grain filling stage.

Figure 3 shows the corresponding channel path loss at different antenna heights in the tillering stage (A) jointing stage (B), and grain filling stage (C). As the distance between the transmitter end and the receiver end (d) increased, the RSSI value decreased and the color changed from blue to red. At this point, the signal is so weak that the receiver end is unable to detect it. There are large differences in the path loss at different antenna heights in stages (A), (B), and (C), but there are similar variation trends (Figure 3). The degree of RSSI attenuation at the three developmental stages showed monotone decreases with changes in antenna height, but the transmission range showed monotone increases with antenna height. These data show that the height of the nodes in the rice field WSN has significant effects on wireless signal transmission. The degree of RSSI attenuation under the four antenna heights had the following order: grain filling stage > jointing stage > tillering stage. The transmission range had the following order: tillering stage > jointing stage > grain filling stage. This shows that, in the growth cycle of rice plants, the transmission environment of the rice field sensor network worsens as the plants grow (reflecting changes in plant height and canopy coverage). At the tillering stage, when the antenna height was 2.0 m, which was taller than rice plants by 1.8 m, RSSI attenuation was the slowest and propagation distance was the furthest. At the grain filling stage, when antenna height was 0.8 m and antenna height was lower than plant height by 0.3 m, RSSI attenuation was the fastest, propagation loss the greatest, and propagation distance was the shortest. This shows that the distance of the node antenna to the plant canopy, crop height, and canopy coverage will affect RSSI attenuation speed and transmission distance.

We used the boundary point of –80 dBm as a reference point for signal intensity since, when the RSSI value decreases to −80 dBm, packet loss occurs. At the grain filling stage when the propagation environment was the worst, at an antenna height of 0.8 m, the propagation distance was 105 m. When the antenna heights were 1.2 m, 1.6 m, and 2.0 m, the propagation distances were 189 m, 266 m, and 301 m, respectively. Therefore, the transmission distance increased by 196 m when the node height increased from 0.8 m to 2.0 m. When node antenna height was increased from 0.8 m to 1.2 m, propagation distance increased by 84 m, which accounted for 42.86% of the total distance increase. When node antenna height was increased from 1.2 m to 1.6 m, propagation distance increased by 77 m, which was 39.28% of the total distance increase. When node antenna height was increased from 1.6 m to 2.0 m, the propagation distance increased by 35 m, which accounted for 17.86% of the total distance increase. These data show that, as node antenna height increases, the increase in propagation distance gradually decreased and wireless propagation distance will not show unlimited increase with increasing antenna height. Therefore, from consideration of communication quality, project implementation, and economic savings, the optimal height for rice field WSNs is approximately 2.0 m from the ground surface.

### 4.2. Path Loss Model Results and Analysis

#### 4.2.1. Free Space Model Results and Analysis

**Figure 4:**
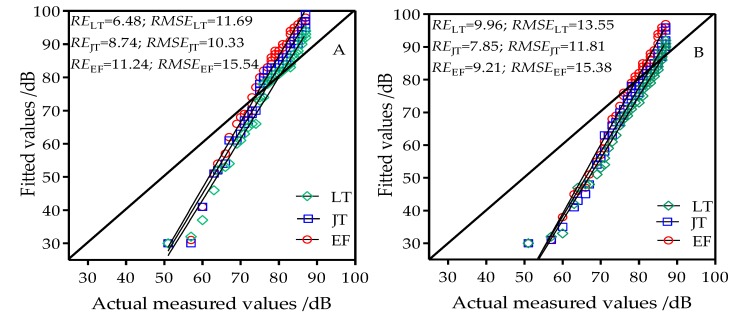

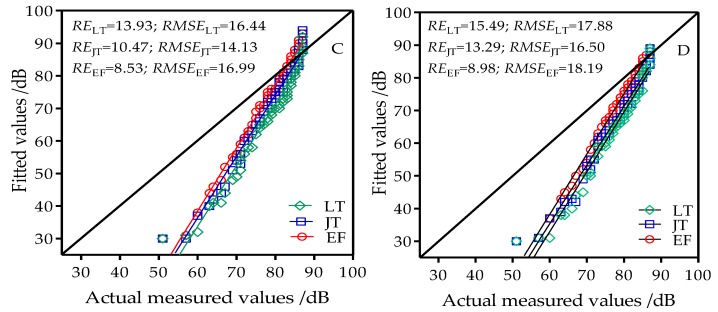
Comparison chart of fitted values from the free space model and actual measured values at antenna heights of (**A**) 0.8 m; (**B**) 1.2 m; (**C**) 1.6 m; and (**D**) 2.0 m; LT: tillering stage; JT: jointing stage; EF: grain filling stage.

The free space model was used to estimate the wireless channel path loss in rice fields in three different developmental stages and under four different heights. Figure 4 shows the rice field performance of the free space model in estimating path loss in wireless channels. When the node antenna heights were 0.8 m, 1.2 m, 1.6 m, and 2.0 m, the performance of the free space model in the three developmental stages was poor, with estimated RE and RMSE values of 6.48–15.49% and 10.33–18.19, respectively. The free space model therefore cannot be directly used for estimation of wireless channel path loss in rice fields. This model was constructed under ideal conditions in which path loss is only related to wireless signal frequency and propagation distance. Therefore, when constructing models for wireless channels in the rice field sensor network, the effects of wireless signal frequency and propagation distance on channel loss must be considered as well as the effects of other factors.

#### 4.2.2. Two-Ray Model Results and Analysis

**Figure 5:**
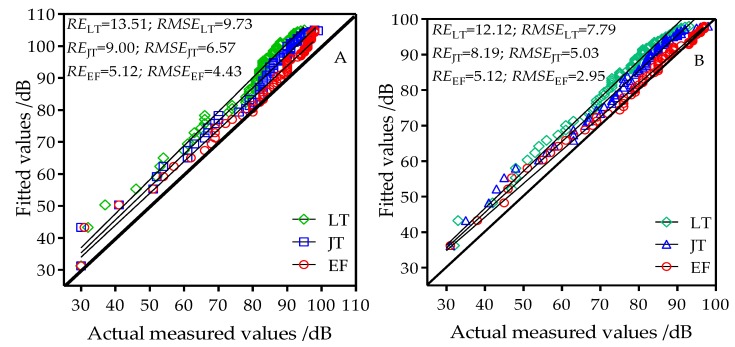

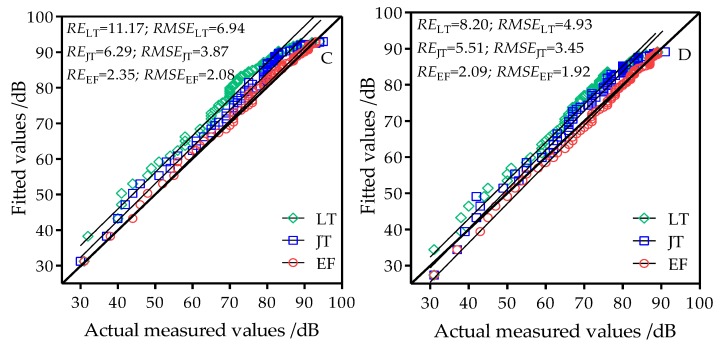
Comparison chart of fitted values from the two-ray model and actual measured values at antenna heights of (**A**) 0.8 m; (**B**) 1.2 m; (**C**) 1.6 m; and (**D**) 2.0 m; LT: tillering stage; JT: jointing stage; EF: grain filling stage.

The two-ray model was used to estimate the wireless channel path loss in rice fields in three developmental stages and four antenna heights. Figure 5 shows the performance of the two-ray model in estimating path loss in rice field wireless channels. Under the antenna heights of 0.8 m, 1.2 m, 1.6 m, and 2.0 m, the estimated RE and RMSE of the tillering stage, jointing stage, and grain filling stage in the two-ray model decreased with increasing node antenna height. At the tillering stage when antenna height was increased from 0.8 m to 2.0 m, RE decreased from 13.51% to 8.20% and RMSE decreased from 9.73 to 4.93. At the jointing stage when antenna height increased from 0.8 m to 2.0 m, RE decreased from 9.00% to 3.45% and RMSE decreased from 6.57 to 1.92. At the grain filling stage when antenna height increased from 0.8 m to 2.0 m, RE decreased from 5.12% to 2.09% and RMSE decreased from 4.43 to 1.92. This shows that, when the two-ray model was used, the node antenna height had a greater effect on model results. As the antenna height changed, the distance between the node antenna and crop canopy also changed. This changed the reflection and transmission distance of the signal through the crop canopy and affected the precision of the estimation using the two-ray model.

The estimated RE and RMSE using the two-ray model decreased with increased rice developmental stage. When the antenna height was 0.8 m, the estimated RE and RMSE decreased from 13.51% and 9.37 to 5.12% and 4.43, respectively, from the tillering stage to the grain filling stage. When the antenna height was 1.2 m, the estimated RE and RMSE decreased from 12.12% and 7.79 to 5.12% and 2.59, respectively, from the tillering stage to the grain filling stage. When the antenna height was 1.6 m, the estimated RE and RMSE decreased from 11.17% and 6.94 to 2.35% and 2.08, respectively, from the tillering stage to the grain filling stage. When the antenna height was 2.0 m, the estimated RE and RMSE decreased from 8.20% and 2.09 to 4.93% and 1.92, respectively, from the tillering stage to the grain filling stage. In the two-ray model, the progress of the developmental stage had a greater effect on the model results. The main reason for this is that the two-ray model considers the combined effects of direct transmission and reflection on channels. As rice plants grow, the reflection caused by changes in plant height and canopy coverage had greater effects on channels. At the tillering stage, plant height and coverage were relatively low and wireless signals are mainly affected by ground reflection. At the jointing stage, wireless signals are also affected by reflection caused by plants in addition to ground reflection. During the grain filling stage, plant growth is more luxuriant and plant reflection is stronger compared to earlier growth stages and reflection effects are more significant. Therefore, the applicability of the model will improve. Compared to the free space model, the two-ray model considers the effects of direct waves, reflected waves caused by crops, and node height on signal transmission in addition to the foundation of wireless signal frequency and propagation distance. When antenna heights were 1.6 m and 2.0 m, the estimated RE and RMSE were all lower than 3.0. This shows that the two-ray model has utility for rice field environments at antenna heights greater than 1.2 m.

#### 4.2.3. One-Slope Log-Distance Model Results and Analysis

The least squares method was used for regression analysis of measured path loss based on Equation (6). Table 1 shows the regression parameters of the one-slope log-distance model for measured path loss at different developmental stages. 

In Table 1, the path loss factor *n* increases with decreasing antenna height, indicating that the transmission environment worsens with decreasing antenna height. At the same antenna height, *n* differs in different developmental stages but all stages showed similar variation trends that were increases with the advance of developmental stages. With developmental stage progression, rice plant height and canopy coverage increase, the canopy layer becomes more luxuriant and the superposition of leaf layers increases. The reflection, refraction, and diffraction of wireless channels will intensify with changes in plant height, reducing the wireless transmission environment of rice fields and increasing path loss factors.

**Figure 6:**
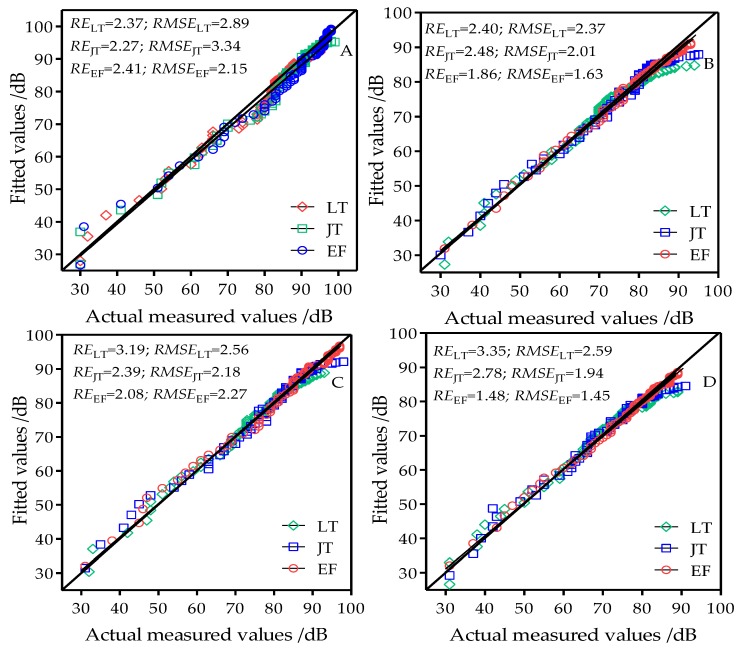
Comparison chart of fitted values from the one-slope log-distance model and actual measured values at antenna heights of (**A**) 0.8 m; (**B**) 1.2 m; (**C**) 1.6 m; and (**D**) 2.0 m; LT: tillering stage; JT: jointing stage; EF: grain filling stage.

A one-slope log-distance model based on Table 1 data was used to estimate the wireless channel path loss in rice fields in three developmental stages and at four antenna heights. Figure 6 shows the performance of the one-slope log-distance model in estimating path loss in wireless channels in rice fields. When the node antenna heights were 0.8 m, 1.2 m, 1.6 m, and 2.0 m, the ranges of the estimated RE and RMSE of the three developmental stages in the one-slope log-distance model were 2.40–2.25% and 3.34–1.45, respectively. These were significantly less than the free space model and the two-ray model. This indicates that the one-slope log-distance model has better performance than the free space model and the two-ray model and has better applicability in modeling of wireless channels in rice fields. However, when the antenna heights were 0.8 m and 1.2 m, the estimated curves in the model were slightly higher or lower than the measured values, but these errors were small. When antenna heights were 1.6 m and 2.0 m, the RE at the tillering stage were all >3.0. This shows that the model has a degree of error when used for estimating path loss in rice fields.

#### 4.2.4. Modified Two-Slope Log-Distance Model Results and Analysis

Least squares regression analysis of measured loss was performed according to Equations (7) and (8). Table 2 shows the regression parameters for the modified two-slope log-distance model in different developmental stages. The results showed that, under the four antenna heights (0.8 m, 1.2 m, 1.6 m, and 2.0 m), the path loss factors of the three developmental stages before the breakpoint were all lower than the path loss factors after the breakpoint. The loss factors obtained from the modified two-slope log-distance model were all larger than the loss factors obtained from the free space model (*n* = 2).

**Figure 7:**
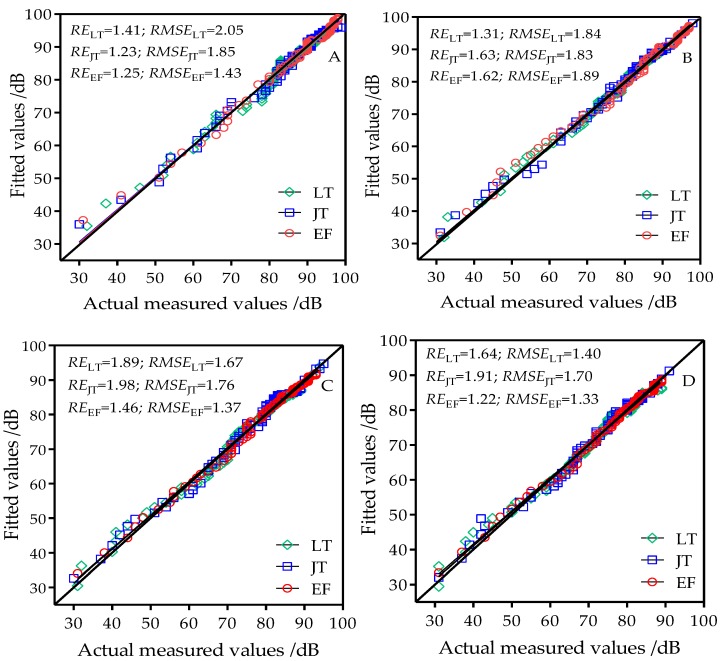
Comparison chart of fitted values from the modified two-slope log-distance model and actual measured values at antenna heights of (**A**) 0.8 m; (**B**) 1.2 m; (**C**) 1.6 m; and (**D**) 2.0 m; LT: tillering stage; JT: jointing stage; EF: grain filling stage.

The modified two-slope log-distance model based on model regression parameters (Table 2) was used to estimate the wireless channel path loss in rice fields at three developmental stages and four antenna heights. Figure 7 shows the performance of the two-slope log-distance model in estimating the path loss of wireless channels in rice fields. When the node antenna heights were 0.8 m, 1.2 m, 1.6 m, and 2.0 m, the ranges of the estimated RE and RMSE of the three developmental stages were 1.89–1.31% and 2.05–1.33, respectively. These values were significantly reduced compared with the free space model, two-ray model, and one-slope log-distance model. Under the four antenna heights, the estimated RE values of the two-slope log-distance model in the three developmental stages were all less than 2%. With the exception of the 0.8 m antenna height in which the estimated RMSE of the modified two-slope log-distance model at the tillering stage was 2.05, the estimated RMSE values at other antenna heights and developmental stages were all less than 2. This shows that the application of the modified two-slope log-distance model in rice fields had better applicability in estimating path loss. The modified two-slope log-distance model had better estimation precision than the one-slope log-distance model and appears more suitable for wireless channel propagation path loss analysis and modeling in rice fields.

## 5. Discussion

The application of WSN in agriculture should consider the interference caused by plant height, leaves, and canopy coverage on channel transmission and effects on data transmission rate and channel loss [25]. We found that the degree of RSSI attenuation under four antenna heights had the following order: grain filling stage > jointing stage > tillering stage. The height transmission range had the following order: tillering stage > jointing stage > grain filling stage. These data show that an increase in the developmental stage had a great effect on model estimation results. Li et al. [13] evaluated attenuation speed acceleration of 2.4 GHz wireless channel propagation and related this to expected progress with crop development. They concluded that the intensification of channel loss was caused by the continuous increase in plant height and stems and leaves becoming more luxuriant as the crop grew and developed. This caused changes in the signal propagation environment when wireless sensor networks are used in farmland and propagation loss can significantly increase. Therefore, the effects of crop growth on network performance should be considered when establishing wireless sensor network nodes in rice fields. When the free space model was used for path loss modeling of 2.4 GHz wireless channels in rice fields, the range of estimated RE and RMSE at different heights is very large. The free space model only considers the effects of wireless signal frequency and propagation distance on channel propagation loss and does not consider the effects of plant height, planting density, stems and leaves in the wireless propagation environment on propagation loss in rice fields. Therefore, the free space model cannot be directly used for analysis of path loss in farmland wireless channels. This conclusion is consistent with the results of Zhang and Zhang [14].

When the two-ray model was used for path loss modeling, the RE and RMSE between the estimated values and measured values at the three developmental stages gradually decreased with plant growth progression. RE and RMSE decreased as node antenna height increased. This shows that rice growth caused significant reflection of propagation channels and changing node antenna height can decrease the estimation precision of the model. Mou et al. [26] showed that as plants grow and the canopy changes, the height distance between the transmitter end and receiver end also changes. Crop cover causes irregular reflection, absorption, and scattering. We found that the lower the node antenna height, the greater the RE and RMSE difference between estimated values and measured values. This is because the two-ray model only considers the effects of height, reflection, and distance on channel loss and neglects the effects of scattering and diffraction. Therefore, the two-ray model cannot be directly used for wireless channel path loss modeling in rice fields.

Ma et al. [27,28] found that, in addition to reflection of wireless channels, crop plants can also cause scattering and diffraction. This further increases signal attenuation at propagation paths, causing the channel transmission environment to gradually worsen. Du et al. [29] found that fitting the measured energy consumption and attenuation using least squares and construction of a one-slope log-distance model using fitting parameters is more suitable for wireless sensor network channel modeling in large fields. When we used the one-slope log-distance model for path loss modeling, RE and RMSE were significantly reduced compared with the free space model and the two-ray model. This is primarily because the one-slope log-distance model comprehensively considers the effects of plant growth changes in rice fields on channel propagation, and path loss factors are obtained from fitting of measured values to construct a channel model. This has better applicability for wireless channel modeling in rice fields and is consistent with previous studies [30,31,32]. However, the estimated RE of the one-slope log-distance model still exceeds 3% at different antenna heights. This shows that some error in path loss remains when this model is used in rice fields. When the least squares method was used for regression analysis of measured loss, we found that the path loss factors during the three developmental stages before the breakpoint were all smaller than those after the breakpoint. The Fresnel zone radius of channels increases as the propagation distance increases. After a certain propagation distance is reached (the breakpoint), crop height and canopy in the field will cause the area of the Fresnel zone to increase to a threshold value that reduces communication quality. This caused the attenuation speed of the wireless signals to increase and was manifested as an increase in path loss factors after the breakpoint. Path loss factors after the breakpoint were greater than path loss factors before the breakpoint. This result is consistent with that obtained by Zhang Haihui [14] in the analysis of the path loss modeling method of a 2.4 GHz wireless channel in a wheat field environment. When the modified two-slope log-distance model was used for modeling path loss, the estimated RE of the two-slope log-distance model in the four antenna heights and three developmental stages were all less than 2%. By setting of breakpoints in the model, the modified two-slope log-distance model increased the precision of the one-slope log-distance model and improved the error produced by the one-slope log-distance model for modeling wireless channels in rice fields. Compared with the one-slope log model, the modified two-slope log-distance model had better applicability for channel propagation in rice fields. Compared with the framework for knowledge discovery from wireless sensor networks in rural environments design developed by Alfonso Gonz Á Lez-Briones [33], this study only constructs a rice field wireless channel model from the perspective of path loss, and should also comprehensively consider and compare different communication protocols and energy conservation and consumption reduction when designing a rice field wireless sensor network. Existing models with consideration of the effects of the aforementioned factors can be used to further improve model precision. This can provide a basis for the engineering, application and networking and node layout for wireless sensor networks in rice fields. In the future, large-scale networking experiments should be conducted to test the performance of the double fold line logarithmic distance model in rice fields. Testing the performance of the wireless sensor network, as an important step before applying the network in precision irrigation, will be our next work. 

## 6. Conclusions

We conducted channel loss tests in rice fields by comparing wireless channel propagation variation trends at four antenna heights and three rice developmental stages. The degree of RSSI attenuation showed monotone decreases with changes in antenna height, but the transmission range showed monotone increases with antenna height. The effects of different node height on wireless channel propagation characteristics in rice fields were significant. The transmission environment of wireless channels in rice field sensor networks deteriorated with developmental stage increase. The optimal height for node antennas was 2.0 m above ground. The free space model is unsuitable for the estimation of path loss in wireless channels in rice fields. The two-ray model can be used in rice fields when antenna height exceeds 1.2 m because height has a large effect on estimation results using the two-ray model. The one-slope log-distance model had good applicability for wireless channel modeling in rice fields, but the estimated RE in the model was >3%. The modified two-slope log-distance model increased the performance of the one-slope log-distance model. The estimated RE of the two-slope log-distance model under four heights was <2% and it had better applicability for the complex environment of rice fields. This model can provide a basis for modeling of sensor network channels and construction of wireless sensor networks in rice fields.

## Figures and Tables

**Table 1:** One-slope log-distance model regression parameters.

Developmental Stage	Parameter *K*				Path Loss Factor, *n*			
	0.8 m	1.2 m	1.6 m	2.0 m	0.8 m	1.2 m	1.6 m	2.0 m
Tillering stage	−5.30	−13.08	−15.31	−15.23	3.79	3.67	3.62	3.58
Jointing stage	−4.94	−13.59	−12.92	−11.78	3.93	3.79	3.75	3.65
Grain filling stage	−4.89	−15.91	−12.07	−9.86	4.19	3.93	3.84	3.66

**Table 2:** Regression parameters of the modified two-slope log-distance model.

		Parameter, *K*				Path Loss Factor, *n*				Breakpoint Distances, *d*/m			
		0.8 m	1.2 m	1.6 m	2.0 m	0.8 m	1.2 m	1.6 m	2.0 m	0.8 m	1.2 m	1.6 m	2.0 m
**Tillering Stage**	**Before Breakpoint**	−8.89	−8.98	−6.70	−7.72	3.87	3.57	3.25	3.24	231	238	168	203
	**After Breakpoint**	−26.71	−57.51	−53.38	−62.21	4.47	5.54	5.25	5.52				
**Jointing Stage**	**Before Breakpoint**	−12.78	−0.94	−3.36	−3.36	3.47	3.00	3.14	3.09	189	154	112	98
	**After Breakpoint**	2.67	−10.14	−16.45	−29.48	4.26	3.80	3.91	4.30				
**Grain Filling Stage**	**Before Breakpoint**	−12.13	−15.09	−4.84	−4.96	3.02	4.15	3.40	3.35	140	147	112	119
	**After Breakpoint**	17.46	−23.67	−18.81	-10.93	4.31	4.50	4.12	3.71				
